# Medical Nutrition Therapy Adherence and Lifestyle in Stage 5 CKD: Challenges and Insights

**DOI:** 10.3390/nu17193091

**Published:** 2025-09-28

**Authors:** Patrizia Palumbo, Gaetano Alfano, Francesca Cavani, Rossella Giannini, Roberto Angelo Pulizzi, Silvia Gabriele, Niccolò Morisi, Floriana Cannito, Renata Menozzi, Gabriele Donati

**Affiliations:** 1 Division of Metabolic Diseases and Clinical Nutrition, Azienda Ospedaliero Universitaria di Modena, 41125 Modena, Italy; 2 Nephrology, Dialysis and Kidney Transplant Unit, Azienda Ospedaliero-Universitaria di Modena, 41125 Modena, Italy; 3 Surgical, Medical, Dental and Morphological Sciences Department (CHIMOMO), University of Modena and Reggio Emilia, 41125 Modena, Italy

**Keywords:** CKD, chronic kidney management, medical nutrition therapy, diet adherence

## Abstract

Background: Adherence to Medical Nutrition Therapy (MNT) is a key determinant of therapy success, particularly in chronic diseases like chronic kidney disease (CKD). MNT in CKD requires significant changes in patient’s dietary habits, which can affect long-term adherence. This study aims to evaluate the adherence to MNT in stage 5 CKD patients undergoing conservative kidney management (CKM), identifying potential challenges and strengths of nutritional intervention. Methods: We enrolled in 94 stage 5 CKD patients undergoing CKM at the University Hospital of Modena, Italy. We collect clinical data from medical and nutrition records. The inclusion criteria comprised patients of all genders, ages, and ethnicity with stage 5 chronic kidney disease (CKD), in pre-dialysis, enrolled in the nephrology and dietetics program, who had access to 24-h urine tests, anthropometric measurements, and dietary history records. Exclusion criteria included patients with CKD stages lower than 5, those who had not undergone at least one nutritional assessment, or lacked accessible 24-h urine data. The study utilized medical and dietary records from September 2017 to March 2025. The primary outcome was the assessment of adherence to medical nutrition therapy (MNT), comparing prescribed protein intake with actual intake, estimated from dietary history (DH). Protein intake was compared with normalized protein nitrogen appearance (nPNA) as stated by recent guidelines. Additional factors influencing adherence, such as age, gender, comorbidities, physical activity, and prior dietary interventions, were also evaluated. Anthropometric measurements and biochemical tests were collected, and dietary intake was assessed using a seven-day DH. Results: Data were analyzed using descriptive statistics, linear correlation models, univariate logistic regression, *t*-tests, paired *t*-tests, and chi-square tests, with significance set at *p* < 0.05. Most of the patients follow suggested energy and protein intakes limits; however, substantial individual variability emerged Bland–Altman analysis indicated a moderate bias and wide limits of agreement for energy intake (+116 kcal; limits of agreement –518.8 to +751.3 kcal), revealing frequent overestimation in self-reports. Protein intake showed less systematic error, but discrepancies between dietary recall and biochemical markers persisted. Protein intake decreased significantly over time (*p* < 0.001), while correlation with nPNA did not reach statistical significance (ρ = 0.224, *p* = 0.051). No significant associations were identified between adherence and most clinical or lifestyle factors, although diabetes was significantly associated with lower adherence to protein intake (*p* = 0.042) and a predominantly sedentary lifestyle showed a borderline association with energy intake adherence (*p* = 0.076), warranting further investigation. Longitudinal analysis found stable BMI and body weight, alongside notable reductions in sodium (*p* = 0.018), potassium (*p* = 0.045), and phosphorus intake (*p* < 0.001) over time. Conclusions: Assessing dietary adherence in CKD remains complex due to inconsistencies between self-reported and biochemical estimates. These findings highlight the need for more objective dietary assessment tools and ongoing, tailored nutritional support. Multifaceted interventions—combining education, personalized planning, regular monitoring, and promotion of physical activity—are recommended to enhance adherence and improve clinical outcomes in this vulnerable population.

## 1. Introduction

**Figure 1:**
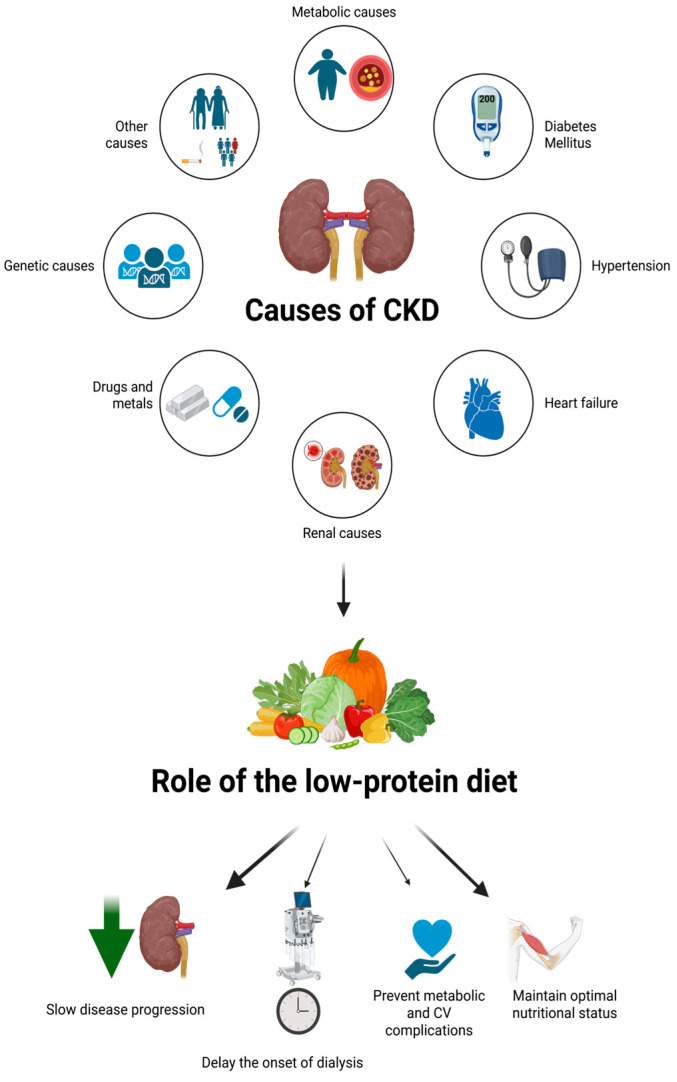
The figure illustrates key renal etiologies of CKD, highlighting their contribution to progressive nephron loss. A low-protein nutritionally balanced diet is emphasized as a non-pharmacological strategy to mitigate renal workload, delay dialysis initiation, and preserve metabolic and nutritional homeostasis. Created in https://BioRender.com, accessed on 25 September 2025.

Chronic Kidney Disease (CKD) is a chronic degenerative disease defined as the presence of anomalies of kidney structure and function for at least 3 months. This condition can have serious health implications, including an increased risk of cardiovascular disease, anemia, bone disorders, and a decline in overall quality of life. Early detection and management are crucial to slowing the progression of CKD and mitigate these adverse outcomes [1]. Notably, the prevalence of CKD in Western countries has risen in step with increasing life expectancy, reaching 30% of the population over the age of 70 [2]. While the prevalence of glomerulonephritis, Autosomal Dominant Polycystic Kidney Disease (ADPKD), and interstitial nephropathies has remained relatively stable in recent years, hypertension and diabetes continue to increase and contribute to progression to end-stage renal disease (ESRD) [3,4,5]. Studies have demonstrated that appropriate Medical Nutrition Therapy (MNT) can delay the need for renal replacement therapy (RRT) in patients still in conservative kidney management (CKM), as shown in Figure 1 [6,7,8,9]. Additionally, regular physical activity has been shown to provide numerous benefits for CKD patients, including improved physical function, enhanced cardiometabolic and neuromuscular function, cognitive benefits, and an overall improvement in quality of life [10,11].

Understanding how nutrition and physical activity impact CKD progression and patient outcomes has been well-investigated in the past. Adherence to MNT is crucial for the success of treatment, especially in chronic diseases like CKD [1,12,13,14,15,16,17,18,19,20,21,22]. In fact, studies have highlighted the effect of different levels of protein intake, depending on the CKD stage, but a significant variation in adherence rates among patients remains [1,2,6,7,12,13,14,17,20,21,23,24,25]. These differences in evidence, underscore the needs of adapting the dietary plans, but mostly of improving the continuous education of patients [25].

Despite advancements in understanding the role of nutrition in CKD management, not many studies have investigated the effective adherence to MNT in patients with CKD. This gap is particularly evident in stage 5 CKD patients undergoing conservative kidney management, a clinically fragile and underrepresented population.

To address this gap, the present retrospective, monocentric study aims to evaluate the adherence to MNT in stage 5 CKD patients undergoing conservative kidney management identifying potential challenges and strengths of the nutritional intervention. Through a longitudinal design that integrates dietary history (DH), biochemical markers, and clinical variables—including behavioral and metabolic factors such as diabetes and physical activity—this study offers a nuanced perspective on nutritional adherence in advanced CKD.

## 2. Materials and Methods

### 2.1. Study Design and Population

In this observational, monocentric, retrospective, non-interventional study, we analysed consecutive adult patients who were treated at the Nephrology, Dialysis and Kidney Transplant Unit, and at the Division of Metabolic Diseases and Clinical Nutrition at the University Hospital of Modena, Italy from September 2017 to March 2025.

Patients of all ages, genders, and ethnicities with stage 5 CKD, in CKM have been enrolled. To participate in the study, patients had to be in charge in the outpatient nephrology and dietetics follow-up program, meaning they needed to be treated by the nephrology team and the specialized nutritional team involved in the management of chronic kidney disease. Furthermore, they had to provide 24-h urine test results, anthropometric measurements, and a comprehensive dietary history (DH) evaluated with Nutrition Care Process Terminology (NCPT) by a specialized nephrology dietitian [26]. Exclusion criteria included individuals at a CKD stage < 5, patients who had not undergone at least one nutritional assessment, or those without accessible 24-h urine test data.

Information regarding personal and social history, including sex, age, and employment status, were obtained from medical and nutritional records. Patients were classified into three occupational categories: retirees/unemployed/students, workers, and other. Data about patient health history, such as the origin of CKD and the presence of clinical and metabolic comorbidities, including hypertension, diabetes, hyperuricemia, dementia, obesity, and dyslipidemia have been also collected.

The study was approved by the Ethics Committee of the Northern Emilia Area (AVEN) (Protocol No. 507/2021/OSS/AOUMOP, SIRER ID 2845) on 7 November 2024, and all participants provided their written informed consent to participate.

### 2.2. Anthropometric Measurements

Anthropometric data were collected from dietary records, including weight (measured with a floor scale with 0.1 kg sensitivity), height (measured with a stadiometer with 0.01 m sensitivity), and BMI categorized according to WHO guidelines.

### 2.3. Biochemical Tests, Clinical Tests, and Procedures

The following laboratory analyses were performed: electrolytes and renal profile (eGFR CKD-EPI, creatinine, phosphorus, sodium, calcium, potassium, PTH, urea), glucose/endocrine profile (glucose, HbA1c), urinary profile (24-h urine urea, proteinuria, sodium, potassium, creatinine clearance, albuminuria), gastrointestinal profile (uric acid), and protein profile (albumin).

### 2.4. Nutritional and Lifestyle Assessment

Nutritional intake was assessed using DH defined as a comprehensive assessment tool used to evaluate an individual’s usual food intake and eating patterns over an extended period. This method involves an in-depth interview conducted by a trained dietitian, to gather information about the types and quantities of foods consumed, meal frequency, and dietary habits [27]. In this case the interview focused on the 7 days prior to the assessment, utilizing the Scotti-Bassani photographic atlas or self-reported food diaries [28]. The dietary history included total energy consumption (kcal/day), energy derived from ethanol (kcal/day), average daily alcohol units, total protein intake (g/day), protein intake relative to body weight (g/kg), salt intake from food (mg/day), potassium intake (mg/day), and phosphorus intake (mg/day). Estimated intakes were calculated using the Food Composition Database for Epidemiological studies in Italy [29]. To evaluate the Physical Activity Level (PAL), we adopted the definitions outlined in the LARN IV edition [30].

### 2.5. MNT Prescription

All patients received an individualized low-protein diet according to KDOQI 2020 recommendations for stage 5 CKD under conservative management [25]. The prescribed protein intake was generally within the range suggested by the guidelines for advanced CKD, with adjustments based on nutritional status and comorbidities such as diabetes. Energy intake was set to maintain body weight and prevent protein-energy wasting, typically around 25–35 kcal/kg/day. Sodium intake was restricted, and potassium and phosphorus adjustments were made according to laboratory values. In selected cases, low-protein products or ketoanalogue supplementation were used to help patients meet protein targets without compromising energy intake. Fluid intake was individualized based on urine output and clinical assessment.

### 2.6. Methodological Approach to Assessing Adherence to MNT

All patients received a nutritional intervention with the registration of the DH at the beginning and during the follow-up evaluations. The primary outcome was to assess the cohort’s adherence to MNT by comparing the suggested protein and energy intake to the actual intake estimated using DH conducted by a trained nephrology dietitian. This method provided detailed estimates of daily protein and energy consumption based on patient-reported food intake. To further assess protein intake, normalized protein nitrogen appearance (nPNA), calculated from 24-h urinary urea excretion, was used as a biochemical comparator to validate and complement the dietary data, in accordance with KDOQI 2020 guidelines [25]. Additional characteristics that may affect adherence to MNT, including age, gender, comorbidities, physical activity level, and prior exposure to dietary interventions have been also ascertained.

$$nPNA=\frac{\left[Daily\;diuresis\;\left(mL\right)\times \frac{24\;h\;Urinary\;Urea\;(g/24\;h)}{2.03}\right]+[Body\;weight\;(Kg)\times 0.031]}{Body\;Weight\;(Kg)}\;\times \;6.25$$

### 2.7. Statistical Analysis

All analyses were performed using IBM SPSS Statistics version 31 (IBM Corp., Armonk, NY, USA). Normality of continuous variables was assessed using the Shapiro–Wilk test, which indicated non-normal distribution (*p* = 5.65 × 10^−6^); therefore, all continuous data were expressed as median and interquartile range (IQR: Q1–Q3), and non-parametric methods were applied.

Descriptive statistics summarized baseline characteristics and dietary intake estimates. Categorical variables were reported as absolute frequencies and percentages. Associations between DH data and biochemical estimates (nPNA and energy expenditure) were evaluated using Spearman’s rank correlation coefficient, due to the non-normal distribution of variables.

Longitudinal changes between the first and last dietetic visit were assessed using the Wilcoxon signed-rank test for paired samples. This included comparisons of BMI, energy intake, protein intake, protein intake per kg of ideal body weight, and intake of sodium, potassium, phosphorus, and alcohol.

Agreement between dietary estimates and prescribed values was further examined using Bland–Altman analysis for both total energy intake and protein intake normalized to reference body weight.

Trends in clinical and nutritional indicators over time were investigated using linear mixed models, focusing particularly on changes in BMI and urinary sodium excretion.

Finally, univariate and multivariate logistic regression analyses were conducted to identify potential predictors of adherence to recommendations for energy intake, protein intake, and nPNA. All tests were two-sided, and statistical significance was set at *p* < 0.05.

## 3. Results

### 3.1. Characteristics of Patients

All the data were collected for 94 patients undergoing pre-dialysis CKM, who were followed at the University hospital of Modena, Italy from September 2017 to March 2025 patients. The population comprised 55 men (58.5%) and 39 women (41.5%). Ages ranged from 43 to 90 years, averaging 68 77 years (IQR: 68–83). The most common causes of CKD were diabetic nephropathy (27.7%), and nephroagiosclerosis (26.6%). Clinical and metabolic complications included hypertension (99.5%), diabetes mellitus (49.2%), hyperuricemia (78.3%), dyslipidemia (61.5%), and dementia (6.4%). Pharmacological treatments included antihypertensives (98.9%), hypolipidemic treatments (72.4%), antigout agents (78.7%), calcitriol (60.9%), hypoglycemic agents (53.9%), sodium bicarbonate (45.1%), calcium carbonate (15.6%), and non-calcic phosphate binders (4.5%). Characteristics of patients are described in Table 1.

### 3.2. Lifestyle Characteristics

We analysed the employment of patients involved in the study, and it emerged that 83.7% were retirees/students/unemployed, 3.4% were office workers, and 13.0% had other jobs. As previously specified, we utilized the definitions from the LARN IV edition to classify physical activity levels [30]. The results indicated that 10.1% of participants were sedentary, 71.0% were predominantly sedentary, and 18.9% were moderately active. The average BMI was 27.70 kg/m^2^ (IQR: 24.30–31.10), with most patients being overweight. During routine follow-up, 25 out of 94 patients (26.6%) died, while 36 patients (38.3%) underwent substitutive treatments: 30.9% started hemodialysis, 5.9% received peritoneal dialysis, and 2.1% underwent kidney transplantation (Table 1).

### 3.3. Nutritional History

Patients received a nutritional follow-up with a median duration of 11.2 months (IQR 5–19.3) with a range of 1–56 months. The median number of nutritional and medical assessments conducted during the follow-up was 6 (IQR: 4–8). Among all the patients involved in the study, 49/94 (47.9%) had previously adhered to a nutritional intervention in other centers, while the remaining 45/94 (52.1%) had never been exposed to any nutritional intervention. Regarding the low-protein diet it was observed that 36 (38.3%) utilized low-protein products to reach the suggested amount of daily protein intake, whereas 4 (4.3%) consumed keto analogues.

### 3.4. Results of Nutritional Adherence Analysis

To assess adherence to the prescribed nutritional plan, two complementary analyses were conducted. The estimated energy and protein intakes reported by patients were compared with the prescribed intakes. Median energy intake at the first visit was 1550 Kcal/day (IQR: 1300–1750), and 1500 Kcal/day (IQR: 1250–1750) at the end of follow-up, while the prescribed intake was 1600 Kcal/day (IQR: 1500–1750). Median protein intake normalized to reference body weight was 0.78 g/kg (IQR: 0.62–0.97) at the first visit and 0.70 g/kg (IQR: 0.58–0.84) at the end of follow-up, while the prescribed intake was 0.80 g/kg (IQR: 0.71–0.88). The Wilcoxon signed-rank test revealed a statistically significant reduction both in absolute protein intake (*p* < 0.001) and in intake normalized to ideal body weight (*p* = 0.002). This indicates a high level of adherence at baseline. Over time, a reduction in protein intake was observed, which may reflect either improved adherence to dietary prescriptions or a natural decline in intake due to disease progression.

Adherence to protein intake was statistically significant (Wilcoxon signed-rank test: Z = −2.492, *p* = 0.013), with DH estimated intake higher intake than prescribed. Protein intake (Z = −4.568, *p* < 0.001) and protein intake normalized to ideal body weight (Z = −3.170, *p* = 0.002) both decreased significantly.

Spearman’s rank correlation analyses showed a weak positive association between reported protein intake (normalized to reference body weight) and nPNA values at both time points: initial (ρ = 0.224, *p* = 0.051, n = 76) and final (ρ = 0.216, *p* = 0.077, n = 68), neither reaching statistical significance.

Similarly, urinary sodium (Z = −2.359, *p* = 0.018), potassium (Z = −2.009, *p* = 0.045), and phosphorus (Z = −3.755, *p* < 0.001) showed significant reductions. These findings suggest a trend toward dietary adjustment over time, particularly in protein and mineral intake. However, reported energy intake and BMI did not change significantly, indicating that overall caloric intake and body weight remained stable.

**Figure 2:**
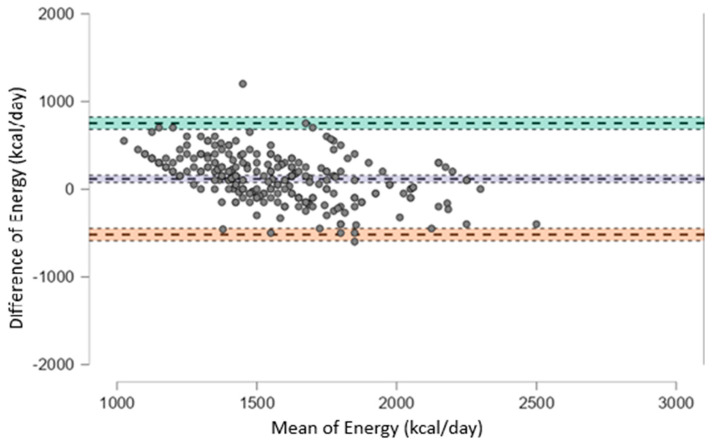
Bland–Altman plot showing the agreement between estimated and prescribed total energy intake (kcal/day). The plot displays the difference between estimated and prescribed values against their mean for each subject. The solid line represents the mean bias (+116.3 kcal/day), while the upper and lower dashed lines indicate the limits of agreement (+751.3 and −518.8 kcal/day, respectively). Wide limits of agreement suggest substantial individual variability between the two methods.

To further evaluate the agreement between dietary estimates and prescribed values, Bland–Altman plots were generated. As shown in Figure 2, for total energy intake, the mean bias was +116.3 Kcal (95% CI: 75.9 to 156.6), with wide limits of agreement ranging from −518.8 to +751.3 Kcal, indicating substantial individual variability and suggesting that the two methods are not fully interchangeable.

Figure 2 illustrates the agreement for protein intake normalized to reference body weight, the mean bias was −0.024 g/kg (95% CI: −0.064 to 0.017), with wide limits of agreement from −0.460 to +0.413 g/kg. Although the systematic difference was negligible, the individual variability may still be clinically relevant, especially in patients with strict protein restrictions.

**Figure 3:**
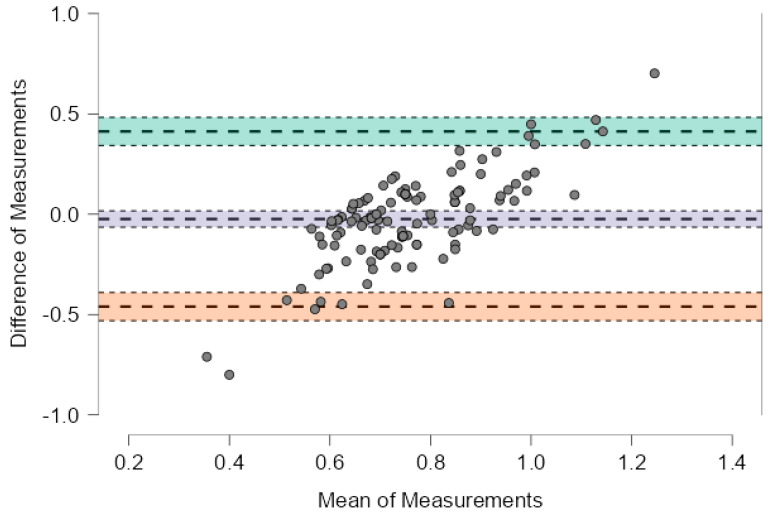
Bland–Altman plot showing the agreement between estimated and prescribed protein intake normalized to reference body weight. The plot displays the difference between estimated and prescribed values against their mean for each subject. The solid line represents the mean bias (−0.024 g/kg), while the upper and lower dashed lines indicate the limits of agreement (+0.413 and −0.460 g/kg, respectively). The systematic difference is minimal, but substantial individual variability is observed.

In Table 2 data are reported related to the comparison between the values at the first and the last dietetic visit. In Table 3 and Table 4 are reported Bland-Altman results, corresponding to Figure 2 and Figure 3 respectively.

### 3.5. Evolution of Dietary and Clinical Indicators over Time

**Figure 4:**
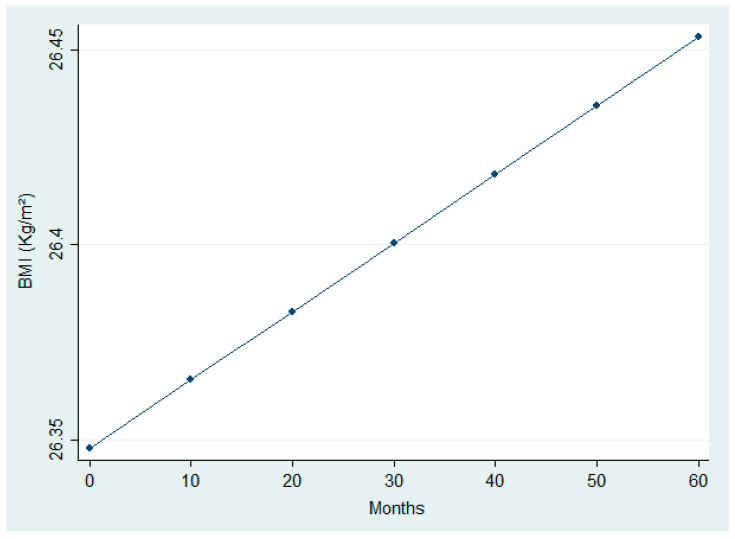
Linear increase in Body Mass Index (BMI) over time.

**Figure 5:**
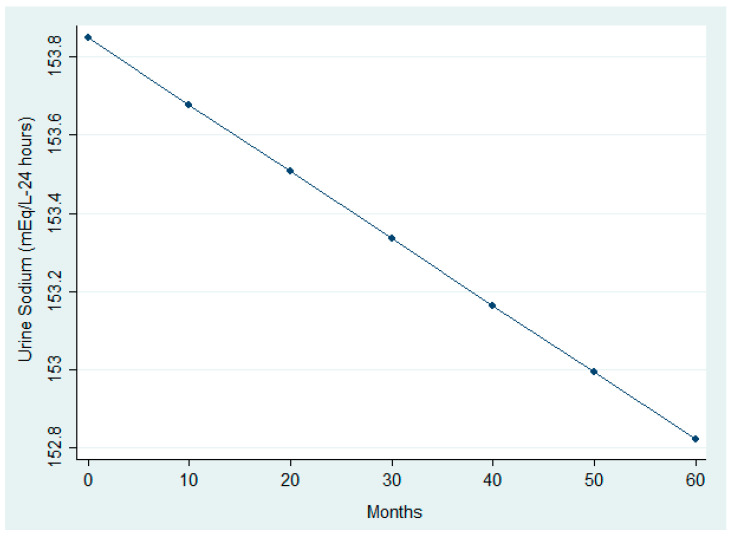
Longitudinal trend in urinary sodium excretion.

The analysis (Table 5 and Table 6) revealed significant modifications in selected clinical and nutritional parameters across follow-up. While paired comparisons between first and last visits did not detect significant BMI changes, the LMM identified a small but statistically significant upward trend over time (β_time = +0.0000587, *p* = 0.022). This trend is visually represented in Figure 4, which shows a linear increase in BMI over time. In addition, Figure 5 illustrates a significant reduction in urinary sodium excretion over time (*p* = 0.031). Age was inversely associated with both urinary sodium (*p* = 0.001) and BMI (*p* = 0.001), whereas male sex was linked to higher urinary sodium levels (*p* = 0.019).

### 3.6. Univariate and Multivariate Logistic Regressions

Univariate and multivariate logistic regression analyses were conducted to explore predictors of adherence to energy intake, protein intake, and normalized protein nitrogen appearance (nPNA) recommendations. None of the models yielded statistically significant predictors at the conventional threshold (*p* < 0.05), although some variables showed trends of interest. In the energy intake multivariate model showed in Table S2 Supplementary Materials, a lifestyle predominantly sedentary was associated with a borderline increase in adherence likelihood compared to completely inactive individuals (OR = 7.8, *p* = 0.076). This may reflect greater nutritional awareness or lower energy requirements among sedentary participants. In the protein intake multivariate model showed in Table S4 Supplementary Materials, diabetes emerged as a significant negative predictor of adherence in the multivariate analysis (OR = 0.34, *p* = 0.042), suggesting that diabetic patients may face greater challenges in meeting protein recommendations. As to adherence measured by means of nPNA, no statistically significant predictors were identified, although higher obesity categories showed elevated odds ratios with wide confidence intervals, indicating potential associations that warrant further investigation (Table S6 Supplementary Materials).

## 4. Discussion

This observational, retrospective cohort study aimed to identify factors influencing adherence of patients with CKM to MNT. It also aimed to identify strengths and limitations of current nutritional strategies, offering insights for improving their effectiveness. Adherence to prescribed regimens is essential for the success of any therapeutic approach, particularly in chronic conditions that require sustained lifestyle changes. Failure to adhere can result in clinical deterioration and increased healthcare costs [31].

Our findings indicate that median energy and protein intakes estimated from dietary history were generally consistent with prescribed targets, although there was considerable individual variability. This alignment between prescribed and reported protein intake, despite variability, represents a positive finding, suggesting that patients were able to follow the prescribed dietary plan. However, when compared with nPNA, the agreement was weaker, with only a modest, non-significant correlation observed. Bland–Altman analysis revealed a systematic bias in energy intake reporting (+116 kcal) and wide limits of agreement (−518.8 to +751.3 kcal), suggesting that self-reported data may not accurately reflect actual consumption. In contrast, protein intake showed a significant decline between the first and last dietary assessment. While median intake at baseline was already close to the prescribed target, the subsequent reduction may reflect increased adherence among patients who initially exceeded recommendations, rather than a general improvement across the entire cohort. This is clinically relevant because protein restriction remains a cornerstone of conservative CKD management, aiming to reduce nitrogenous waste, slow disease progression, and alleviate uremic symptoms [1,25,30].

Beyond these overall trends, longitudinal analysis revealed demographic and lifestyle factors associated with nutritional and clinical outcomes. Specifically, age was inversely related to BMI and urinary sodium excretion, while male sex was linked to higher sodium levels. These observations are consistent with prior evidence showing that aging is associated with reduced lean mass and altered sodium handling, and that sex differences influence dietary intake and renal sodium regulation [32,33,34].

However, when comparing dietary history estimated protein intake with nPNA measurements, Bland–Altman analysis showed a minimal mean bias (−0.024 g/kg) but wide limits of agreement (−0.460 to +0.413 g/kg), highlighting clinically relevant discrepancies at the individual level. These inconsistencies underscore the challenges of accurately assessing protein intake in CKD patients, who must adhere to a restrictive diet requiring substantial changes in both quantity and quality of food. This variability reinforces the need for ongoing nutritional education and objective monitoring tools to complement dietary recalls.

The discrepancy between reported and actual intake may stem from several factors, including differences in interpretation and estimation methods among professionals, which can introduce operator-dependent variability. Additionally, the small sample size and patients’ tendency to overestimate intake—particularly when dietary patterns are highly variable—may have affected accuracy, as previously documented [35]. Collectively, these findings emphasize the importance of continuous nutritional education and support for CKD patients [35].

No significant BMI change was observed between the first and last dietary visits, confirming weight stability over time. However, mixed-model analysis showed a slight upward trend in BMI, which may reflect heterogeneity in the cohort: some patients could have moved toward normal weight, while others—already overweight or obese—may have gained additional weight. This aspect is clinically relevant because it raises the question of whether weight gain represents recovery from undernutrition or progression toward severe obesity. Given the median age of our cohort (77 years), maintaining weight is a protective factor against malnutrition and muscle loss, both associated with adverse outcomes in older adults. Although the average BMI falls within the overweight range according to WHO criteria, it is noteworthy that BMI values below 25 kg/m^2^ and above 35 kg/m^2^ in the elderly are linked to reduced functional capacity and increased mortality [36,37].

As to electrolyte management, significant reductions in sodium, phosphorus, and potassium intake were also observed during the follow-up. Managing these electrolytes is critical in CKD, as disturbances in mineral metabolism are common [38]. Adequate phosphorus control helps prevent calcitriol decline and secondary hyperparathyroidism, which can lead to bone demineralization and vascular calcification, increasing cardiovascular risk as well as patients’ mortality and progression of CKD [38,39,40]. Similarly, potassium restriction reduces the risk of hyperkalemia and related complications such as arrhythmias and muscle weakness [41]. In addition, sodium restriction plays a key role in CKD management, because excessive sodium intake contributes to hypertension, extracellular fluid overload, and increased proteinuria, all of which accelerate CKD progression and elevate cardiovascular risk [25].

Logistic regression analyses did not identify strong predictors of adherence for energy, protein, or nPNA intake. However, some trends were noteworthy. Interestingly, a predominantly sedentary lifestyle was associated with a borderline increase in energy intake compared to complete inactivity (OR = 7.8, *p* = 0.076), suggesting that even minimal physical activity may be linked to better dietary adherence, possibly due to clustering of health-promoting behaviors [42]. Although this association did not reach statistical significance in our sample, it is consistent with existing literature linking physical activity to improved diet quality [1,42,43]. These findings underscore the importance of integrating physical activity promotion into counseling and structured care programs for CKD management.

In contrast, diabetes was significantly associated with lower adherence to protein intake (*p* = 0.042). This finding underscores the complexity of dietary management in diabetic patients with CKD, who must balance protein restriction with glycemic control. Such dual dietary demands may hinder adherence and highlight the need for personalized nutritional strategies and closer follow-up in this subgroup. For nPNA adherence, higher obesity categories showed elevated odds ratios, though with wide confidence intervals, pointing to potential but inconclusive associations.

These findings have direct implications for clinical practice, underscoring the importance of individualized, patient-centered dietary prescriptions. In line with recent evidence from Piccoli et al. [2], who demonstrated that dietary satisfaction and perceived quality of life are key drivers of long-term adherence to low-protein diets in CKD, our results reinforce the value of structured interventions that integrate personalization, education, and psychological support. For patients facing additional challenges such as diabetes, targeted strategies like simplified meal plans, motivational interviewing, and digital tracking tools may help overcome barriers and improve the sustainability of nutritional interventions.

Although the correlation between protein intake estimated from DH and nPNA was weak and did not reach statistical significance, the consistent positive trend suggests that nPNA can still be a useful complementary marker for monitoring adherence to protein restriction. According to KDOQI recommendations, nPNA should support—not replace—dietetic assessment, as its reliability depends on different conditions such as catabolic state, fluid overload, or the use of ketoanalogues [25]. For this reason, nPNA is most informative when discrepancies arise between self-reported intake and clinical or biochemical data.

Evidence on the optimal structure and frequency of dietetic interventions in CKD is limited, although some studies suggest benefits on risk factors when dietitian-led programs are implemented [44]. In our cohort, patients received a median of six medical and dietetic consultations over eleven months, which likely contributed to the observed improvements in protein and mineral intake. These findings support the concept that regular, structured follow-up may enhance adherence, even though the ideal frequency remains to be defined. Future research should explore whether the frequency of visits has a dose–response effect on adherence, including outcomes based on nPNA.

However, the study has some limitations. The retrospective nature of this study and the small sample size may have introduced some bias, particularly regarding data accuracy and completeness, since it relied on previously recorded information. Another limitation was the absence of a control group, which restricted direct comparisons between adherent and non-adherent patients and may have weakened the robustness of our conclusions. However, in patients with stage 5 CKD under conservative management, nutritional therapy is considered essential, and withholding it for the sake of comparison would not be ethically acceptable. This limitation reflects the clinical priority of providing care over experimental design. Furthermore, most participants were already at an advanced stage of disease, and follow-up duration varied considerably—some patients were monitored only briefly, making it difficult to capture long-term adherence patterns. In addition, dietary intake was self-reported, which raises the possibility of misreporting and discrepancies between perceived and actual adherence. Finally, psychological aspects such as motivation or emotional well-being, which are known to influence dietary behavior, were not thoroughly explored.

Despite these limitations, the findings of the present study carry out important clinical implications. They emphasize the need for frequent dietetic consultation and structured follow-up to improve adherence among CKD patients. Such programs should address the specific challenges of this population and ensure regular monitoring of dietary intake and nutritional status, using both dietary history and nPNA to detect and correct non-adherence early. Moreover, involving patients in planning their dietary regimen may enhance adherence by making changes more acceptable and sustainable [45].

## Figures and Tables

**Table 1:** Characteristics of the patients enrolled.

Etiology of CKD		
ADPKD	n (%)	8 (8.5)
Glomerulonephritis	n (%)	5 (5.3)
Congenital monokidney	n (%)	5 (5.3)
Interstitial Nephritis	n (%)	1 (1.1)
Nephroagiosclerosis	n (%)	25 (26.6)
Diabetic Nephropathy	n (%)	26 (27.7)
IgA Nephropathy	n (%)	1 (1.1)
Unknown	n (%)	1 (1.1)
Ischemic Nephropaty	n (%)	4 (4.2)
Other Etiologies	n (%)	18 (19.1)
**Comorbidities**		
Hypertension	n (%)	93 (98.9)
Diabetes	n (%)	39 (41.5)
Dyslipidemia	n (%)	62 (66.0)
Hyperuricemia	n (%)	71 (75.5)
Dementia	n (%)	6 (6.4)
**Drugs**		
Antihypertensives	n (%)	91 (96.8)
Calcium Carbonate	n (%)	11 (11.7)
Antigout	n (%)	69 (73.4)
Hypolipidemic treatments	n (%)	69 (73.4)
Bicarbonate	n (%)	40 (42.6)
Hypoglycemic treatments	n (%)	43 (45.7)
Calcitriol	n (%)	53 (56.4)
Non-calcic Phosphate binders	n (%)	3 (3.2)
**Lifestyle assessment**		
Retired/students/unemployed	n (%)	78 (83.0)
Employees	n (%)	5 (5.3)
Other activity	n (%)	14 (14.9)
Sedentary	n (%)	11 (11.7)
Predominantly sedentary	n (%)	69 (73.4)
Moderately active	n (%)	14 (14.9)
Intense physical activity	n (%)	0 (0)
Underweight	n (%)	0 (0)
Normal Weight	n (%)	29 (30.9)
Overweight	n (%)	25 (26.6)
Class 1 obesity	n (%)	22 (23.4)
Class 2 obesity	n (%)	4 (4.3)
Class 3 obesity	n (%)	4 (4.3)

*Abbreviations: ADPKD = Autosomal Dominant Polycystic Kidney Disease; IgA = Immunoglobulin A.*

**Table 2:** Comparison between the first and last dietetic visit using Wilcoxon signed-rank test. Data are expressed as paired comparisons using Wilcoxon signed-rank test. BMI, body mass index; IBW, ideal body weight.

Variabile	N	Z (Wilcoxon)	*p*-Value
BMI	94	−1.508	0.132
Energy intake	89	−1.322	0.186
Protein intake	89	−4.568	**<0.001**
Protein intake/kg IBW	63	−3.170	**0.002**
Sodium	85	−2.359	**0.018**
Potassium	85	−2.009	**0.045**
Phosphorus	85	−3.755	**<0.001**
Alcohol units	94	−1.570	0.116

*Abbreviations: BMI = Body Mass Index; IBW = Ideal Body Weight.*

**Table 3:** Bland–Altman analysis for the agreement between estimated total energy intake and prescribed values. Bias is expressed as the mean difference (estimated—prescribed), with limits of agreement calculated as mean difference ± 1.96 SD, and 95% confidence intervals (CI) for each value.

Bias & Limits	Point Value	Lower 95% CI
Mean difference + 1.96 SD	751.285	681.383
Mean difference	116.260	75.902
Mean difference −1.96 SD	−518.765	−588.667

*Abbreviations: CI = Confidence Interval; SD: Standard Deviation.*

**Table 4:** Bland–Altman analysis for the agreement between estimated and prescribed protein intake normalized to reference body weight. Bias is expressed as the mean difference (estimated—prescribed), with limits of agreement calculated as mean difference ± 1.96 SD, and 95% confidence intervals (CI) for each value.

Bias & Limits	Point Value	Lower 95% CI
Mean difference + 1.96 SD	0.413	0.343
Mean difference	−0.024	−0.064
Mean difference − 1.96 SD	−0.460	−0.530

**Table 5:** Mixed Linear Model Results for Urinary Sodium.

Variable	Coefficient	*p*-Value
Time	−0.000573	**0.031**
eGFR	−1.666	0.118
Male sex	23.694	**0.019**
Age	−1.618	**0.001**

*Abbreviations: eGFR = estimated Glomerular Filtration Rate.*

**Table 6:** Mixed Linear Model Results for BMI.

Variable	Coefficient	*p*-Value
Time	0.0000587	**0.022**
eGFR	−0.0255	0.49
Male sex	−1.214	0.271
Age	−0.166	**0.001**

*Abbreviations: eGFR = estimated Glomerular Filtration Rate.*

## Data Availability

The dataset includes patient-level clinical information and therefore cannot be shared publicly for privacy and ethical reasons. Any data requests will be evaluated in accordance with institutional policies and GDPR regulations to ensure compliance with data protection standards.

## Supplementary Materials

The following supporting information can be downloaded at: https://www.mdpi.com/article/10.3390/nu17193091/s1, Table S1: Univariate Logistic Regression–Energy Intake Adherence; Table S2: Multivariate Logistic Regression–Energy Intake Adherence; Table S3: Univariate Logistic Regression–Protein Intake Adherence; Table S4: Multivariate Logistic Regression–Protein Intake Adherence; Table S5: Univariate Logistic Regression–nPNA Adherence; Table S6: Multivariate Logistic Regression–nPNA Adherence.
