# Supplementary material for: Medical Nutrition Therapy Adherence and Lifestyle in Stage 5 CKD: Challenges and Insights
Source: Nutrients. 2025 Sep 28;17(19):3091. doi: 10.3390/nu17193091 (PMC12526388; doi:10.3390/nu17193091)
Supplement: Supplementary file 1 [file nutrients-17-03091-s001.zip › nutrients-3858901-supplementary.pdf]

Logistic Regression Analysis Results

Table 1 Supplementary Materials: Univariate Logistic Regression – Energy Intake Adherence

Predictor Variable	B	SE	Wald	df	p-value	OR	95% CI for OR
Age	0.018	0.022	0.667	1	0.414	1.018	—
Sex	-0.697	0.479	2.119	1	0.145	0.498	—
Diabetes	0.029	0.461	0.004	1	0.950	1.029	—
Dementia	0.953	1.120	0.724	1	0.395	2.593	—
Previous Diets	0.585	0.458	1.632	1	0.201	1.795	—
Occupational Status 1	1.668	—	—	1	0.196	—	—
Occupational Status 2	0.119	—	—	1	0.730	—	—
Physical Activity 1	-1.861	1.206	2.381	1	0.123	0.156	—
Physical Activity 2	-0.289	0.640	0.204	1	0.651	0.749	—
Obesity Category 3	—	—	—	1	0.212	9.114	—
Obesity Category 5	—	—	—	1	0.078	14.993	—

Legend: OR = Odds Ratio; CI = Confidence Interval; SE = Standard Error; df = degrees of freedom. Obesity categories: 1 = normal weight, 2 = overweight, 3 = obesity class 1, 5 = obesity class 3. Physical activity: 1 = predominantly sedentary, 2 = moderately active. Occupational status: 1 = office work, 2 = other.

Table 2 Supplementary Materials: Multivariate Logistic Regression – Energy Intake Adherence

Predictor Variable	B	SE	Wald	df	p-value	OR	95% CI for OR
Obesity (Cat. 1)	-0.322	0.900	0.128	1	0.720	0.724	0.124 – 4.226
Obesity (Cat. 2)	-0.697	0.940	0.549	1	0.459	0.498	0.079 – 3.144
Obesity (Cat. 3)	-0.686	0.934	0.539	1	0.463	0.504	0.081 – 3.143
Obesity (Cat. 4)	-1.022	1.507	0.460	1	0.498	0.360	0.019 – 6.905
Obesity (Cat. 5)	0.057	1.610	0.001	1	0.972	1.059	0.045 – 24.839
Sex	-0.874	0.539	2.629	1	0.105	0.417	0.145 – 1.200
Age	0.010	0.031	0.092	1	0.761	1.010	0.950 – 1.073
Dementia	-0.596	1.195	0.249	1	0.618	0.551	0.053 – 5.735
Diabetes	-0.430	0.534	0.646	1	0.422	0.651	0.228 – 1.855
Occupational Status 1	0.251	1.101	0.052	1	0.819	1.286	0.148 – 11.136

Occupational Status 2	-20.941	14914.123	0.000	1	0.999	0.000	—
**Physical Activity 1**	**2.055**	**1.158**	**3.149**	**1**	**0.076**	**7.805**	**0.807 – 75.516**
Physical Activity 2	2.105	1.285	2.681	1	0.102	8.204	0.661 – 101.884

Legend: OR = Odds Ratio; CI = Confidence Interval; SE = Standard Error; df = degrees of freedom. Obesity categories: 1–5 as defined. Physical activity: 1 = predominantly sedentary, 2 = moderately active. Occupational status: 1 = office work, 2 = other.

Table 3 Supplementary Materials: Univariate Logistic Regression – Protein Intake Adherence

**Predictor Variable**	**B**	**SE**	**Wald**	**df**	**p-value**	**OR**
Age	-0.039	0.031	1.596	1	0.206	0.962
Sex	0.357	0.702	0.258	1	0.611	1.429
Diabetes	-1.099	0.760	2.089	1	0.148	0.333
Dementia	22.206	40192.969	0.000	1	1.000	4405840480.503
Previous Diets	0.830	0.762	1.188	1	0.276	2.294
Occupational Status (2)	0.956	1.067	0.802	1	0.371	2.600
Physical Activity (1)	-0.868	1.008	0.741	1	0.389	0.420
Physical Activity (2)	0.811	1.291	0.395	1	0.530	2.250
Obesity (Cat. 5)	22.589	28420.721	0.000	1	0.999	6461899371.405

Legend: OR = Odds Ratio; SE = Standard Error; df = degrees of freedom. Obesity category 5 = obesity class 3. Physical activity: 1 = predominantly sedentary, 2 = moderately active. Occupational status: 2 = other.

Table 4 Supplementary Materials: Multivariate Logistic Regression – Protein Intake Adherence

**Predictor Variable**	**B**	**SE**	**Wald**	**df**	**p-value**	**OR**	**95% CI for OR**
Age	-0.187	0.104	3.219	1	0.073	0.829	0.676 – 1.017
Sex	-2.248	2.167	1.076	1	0.300	0.106	0.002 – 7.387
**Diabetes**	**-4.210**	**2.074**	**4.119**	**1**	**0.042**	**0.015**	**0.000 – 0.866**
Dementia	28.138	40192.970	0.000	1	0.999	1660971798006.710	—
Physical Activity (1)	1.712	2.005	0.729	1	0.393	5.542	0.109 – 282.106
Physical Activity (2)	4.452	2.809	2.511	1	0.113	85.775	0.348 – 21124.027
Occupational Status (2)	0.956	1.067	0.802	1	0.371	2.600	0.321 – 21.047

Obesity (Cat. 4)	4.096	2.415	2.878	1	0.090	60.129	0.529 – 6831.952

Legend: OR = Odds Ratio; CI = Confidence Interval; SE = Standard Error; df = degrees of freedom. Diabetes = presence of diabetes mellitus. Obesity category 4 = obesity class 2. Physical activity: 1 = predominantly sedentary, 2 = moderately active.

Table 5 Supplementary Materials: Univariate Logistic Regression – nPNA Adherence

Predictor Variable	B	SE	Wald	df	p-value	OR
Age	-0.025	0.019	1.675	1	0.196	0.975
Sex	0.059	0.438	0.018	1	0.892	1.061
Diabetes	-0.529	0.450	1.382	1	0.240	0.589
Dementia	-1.050	1.118	0.882	1	0.348	0.350
Physical Activity (1)	0.875	0.822	1.135	1	0.287	2.400
Physical Activity (2)	1.504	0.947	2.523	1	0.112	4.500
Occupational Status (1)	1.157	0.945	1.500	1	0.221	3.180
Occupational Status (2)	0.569	0.652	0.761	1	0.383	1.767
Obesity (Cat. 3)	2.015	1.138	3.136	1	0.077	7.500

Legend: OR = Odds Ratio; SE = Standard Error; df = degrees of freedom. Obesity category 3 = obesity class 1. Physical activity: 1 = predominantly sedentary, 2 = moderately active. Occupational status: 1 = office work, 2 = other.

Table 6 Supplementary Materials: Multivariate Logistic Regression – nPNA Adherence

Predictor Variable	B	SE	Wald	df	p-value	OR	95% CI for OR
Age	-0.020	0.029	0.442	1	0.506	0.981	0.926 – 1.039
Sex	0.026	0.515	0.002	1	0.960	1.026	0.374 – 2.818
Diabetes	-0.293	0.535	0.301	1	0.583	0.746	0.262 – 2.127
Dementia	-0.890	1.182	0.567	1	0.452	0.411	0.040 – 4.168
Obesity (Cat. 3)	2.210	1.200	3.392	1	0.066	9.114	0.868 – 95.721
Obesity (Cat. 5)	2.708	1.536	3.107	1	0.078	14.993	0.739 – 304.352
Physical Activity (2)	1.351	1.058	1.630	1	0.202	3.861	0.485 – 30.711
Occupational Status (1)	0.786	1.065	0.545	1	0.461	2.194	0.272 – 17.689

Legend: OR = Odds Ratio; CI = Confidence Interval; SE = Standard Error; df = degrees of freedom. Obesity categories: 3 = obesity class 1, 5 = obesity class 3. Physical activity: 2 = moderately active. Occupational status: 1 = office work.
